# Mining Primary Care Electronic Health Records for Automatic Disease Phenotyping: A Transparent Machine Learning Framework

**DOI:** 10.3390/diagnostics11101908

**Published:** 2021-10-15

**Authors:** Fabiola Fernández-Gutiérrez, Jonathan I. Kennedy, Roxanne Cooksey, Mark Atkinson, Ernest Choy, Sinead Brophy, Lin Huo, Shang-Ming Zhou

**Affiliations:** 1 Swansea University Medical School, Swansea University, Swansea SA2 8PP, UK; fabiola.fg@gmail.com (F.F.-G.); J.I.Kennedy@Swansea.ac.uk (J.I.K.); r.cooksey@swansea.ac.uk (R.C.); m.atkinson@swansea.ac.uk (M.A.); S.Brophy@swansea.ac.uk (S.B.); 2 Arthritis Research UK CREATE Centre, Division Infection and Immunity, Cardiff University, Cardiff CF10 3NB, UK; ChoyEH@cardiff.ac.uk; 3 Welsh Arthritis Research Network, School of Medicine, Cardiff University, Cardiff CF10 3NB, UK; 4 China-ASEAN Research Institute, Guangxi University, Nanning 530004, China; 19950043@gxu.edu.cn; 5 Centre for Health Technology, Faculty of Health, University of Plymouth, Plymouth PL4 8AA, UK

**Keywords:** phenotyping, rheumatology, cohort identification, electronic health records, feature selection, transparent machine learning, text mining, big data, artificial intelligence

## Abstract

(1) Background: We aimed to develop a transparent machine-learning (ML) framework to automatically identify patients with a condition from electronic health records (EHRs) via a parsimonious set of features. (2) Methods: We linked multiple sources of EHRs, including 917,496,869 primary care records and 40,656,805 secondary care records and 694,954 records from specialist surgeries between 2002 and 2012, to generate a unique dataset. Then, we treated patient identification as a problem of text classification and proposed a transparent disease-phenotyping framework. This framework comprises a generation of patient representation, feature selection, and optimal phenotyping algorithm development to tackle the imbalanced nature of the data. This framework was extensively evaluated by identifying rheumatoid arthritis (RA) and ankylosing spondylitis (AS). (3) Results: Being applied to the linked dataset of 9657 patients with 1484 cases of rheumatoid arthritis (RA) and 204 cases of ankylosing spondylitis (AS), this framework achieved accuracy and positive predictive values of 86.19% and 88.46%, respectively, for RA and 99.23% and 97.75% for AS, comparable with expert knowledge-driven methods. (4) Conclusions: This framework could potentially be used as an efficient tool for identifying patients with a condition of interest from EHRs, helping clinicians in clinical decision-support process.

## 1. Introduction

Identifying patients who satisfy predefined criteria with a particular condition from electronic health records (EHRs) can be used in numerous research studies, such as clinical trial recruitments, survival analysis, or outcome predictions, etc. [1,2]. Importantly, EHRs-based cohorts allows studies on rare conditions or genomic studies to have the potential of scaling to large populations with a sufficiently powered analysis. Larger cohorts are a key contribution to novel discoveries in genetic medicine [3]. But the central question is: how can we define or identify the criteria that accurately describe the condition of interest within the EHRs?

Currently, the patient records held in primary care contain the most comprehensive medical history for a population, including information on symptoms, diagnoses, referrals, treatment, and changes in an individual’s health over time, representing an incredible resource for cohort identification research. However, there are many challenges in using primary care health records to identify cohorts of patients with a particular condition. First, the quality of the records can vary [1]; in particular, the diagnostic codes in primary care may be inaccurate. For example, using medical records in Medicare database (USA), the positive predictive values (PPVs) were 55.7% for at least two claims coded for RA, 65.5% for at least three claims for RA, and 66.7% for at least two rheumatology claims for RA [4]. From the Clinical Practice Research Datalink, a routinely collected primary care database (UK), the accuracy of identifying someone as definite RA by a single RA Read code was 78.7% [5]. A recent study in the Netherlands suggests that more than half of the children with asthma diagnosed and treated by their general practitioners (GPs) may not actually have the condition [6], leading to unnecessary treatment, disease burden, and impact on quality of life and putting them at risk from the side effects of their medication. These findings suggest that some conditions may appear under-reported in primary care and others over-reported. In contrast, patient records held in secondary care contain more robust diagnostic data than primary data, but they are sparse, often contain only severe active disease, and are not easily available. Therefore, an ideal and natural scheme is to link the primary care records with the secondary care ones at patient level, in which the secondary care records act as a gold standard to enhance the accuracy of the codes selected from primary care for a population.

However, distinguishing patients with a particular condition from their records can be extremely time consuming and resource costly. This is because the criteria that characterise a condition, particularly a chronic condition, are buried within complex hierarchical terminology structures across multiple data points in the records of a patient, such as the Read codes [7] used in General Practice in the United Kingdom or the SNOMED CT (Systematized Nomenclature of Medicine Clinical Terms) [8], which merge the SNOMED Reference Terminology and Read codes and is promoted in many countries. These hierarchical terminology structures with extensive overlap of classes lead to a huge number of codes (terms) describing the condition of a patient across multiple data points. In addition, prevalence of a certain condition within a population is comparatively very small. This results in a both highly dimensional (large number of medical codes/characteristics) and imbalanced (few positive patients) EHR data space. All these issues present a big methodological challenge in identifying cohorts from EHRs, making the classical statistical modelling techniques no longer feasible due to the curse of dimensionality [9,10]. Thus, to distinguish patients with a condition in primary care, it becomes crucial to identify the most predictive code patterns buried in their EHRs rather than consider the whole set of overlapping codes. To achieve this, researchers have turned to experts’ review of records to manually select the most relevant codes in safeguarding the accuracy of patient identification [11,12]. However, this methodology is expensive, inefficient, and highly subjective and depends on the local healthcare system and the level of clinicians’ knowledge.

Different from the expert-knowledge-driven methods, AI- and machine-learning-based, data-driven methods offer a promising application in improving diagnostic performance [13,14]. In this paper, we propose to use a transparent machine-learning framework to mine EHRs for automatic phenotype identification by parsimonious set of useful clinical signals. The proposed methodology is grounded in the theory and methods of text mining [15]. Each patient is treated as a document consisting of coded terms and other variables (words); prediction of health outcomes for each patient is thus treated as a problem of text categorisation. One efficient way of representing words in vector space is to embed words in a latent factor vector space [16,17]. Although latent representation of words may significantly improve performance of word classification, it is difficult or impossible to know the logic involved about relationships between the raw words and the outcome. As the General Data Protection Regulation (GDPR) [18] takes effect, data subjects have the right to have meaningful information about the logic involved and the envisaged consequences of such automated decision making for the data subject. It is of pressing importance to develop transparent data-driven models that have explanatory power. This is essential in order for machine-learning-based tools to be sufficiently trusted by medical community in modifying protocols for diagnosis and treatment. Therefore, in this paper, we directly work on raw words rather than their latent representations by integrating data-mining techniques to offer a transparent disease-phenotyping framework that generates decision rules with clear inferencing logic involved about relationships between clinical concepts and health outcome. To reduce the large number of raw words to the minimum combination of features able to phenotype a condition and with each feature representing a single EHR within a patient’s medical history, feature selection (FS) is used to score and rank the clinical codes (terms) so that only the most relevant codes are kept.

Clinical data are commonly imbalanced with a small number of positive patients within a large patient cohort. However, most health statistical analysis studies did not consider the problem of imbalance of clinical data; these studies inherently and inexplicitly put more emphasis on learning data observations with more occurrences. Instead, our proposed methodology suggested to use cost-sensitive machine-learning techniques to tackle this challenge. In this way, this framework enables the patterns examined in the primary care records to be able to identify the presence or absence of the condition as accurately as possible, at best with a secondary care diagnosis.

In this study, two arthritis conditions are used as exemplar of this framework: rheumatoid arthritis (RA) and ankylosing spondylitis (AS). Moreover, we compared this data-driven framework with the standard clinical expert-knowledge-driven algorithms for both RA and AS.

In summary, the contribution of this study includes: (1) the proposed methodology focused on generating transparent knowledge from data; (2) from a large number of factors, a parsimonious set of influential clinical signals with the fewest number of variables were identified while system predictive performance was maintained; (3) the proposed framework worked efficiently with a large and very high dimensional dataset, which allows the predictive models to avoid the challenges of dimensionality; and (4) the proposed framework suggested to use cost-sensitive machine-learning techniques to address the common but ignored challenge of the imbalance of clinical diagnostic data.

## 2. Materials and Methods

### 2.1. Data Mining and Machine-Learning Techniques

In primary care, the terminology structures in EHRs (e.g., Read codes, SNOMED-CT) often generate a huge number of codes (terms) to describe a patient across multiple care points. In order to provide a guideline for selecting influential codes (i.e., features in data analytics), we used five data-mining feature-selection methods to quantify their significance in identifying a particular condition of a patient. The five methods used for this analysis are χ^2^ statistic, Binomial distribution [19], Information Gain (IG) [20], Gini index [21], and DKM index [22].

In this study, decision trees were chosen as classification algorithms within the automatic data-driven model, while other types of machine-learning classifiers can also be used in this framework. We chose decision trees due to its strengths that each path from the root of the tree to each of the leaves (internal or terminal nodes) can be transformed into a rule describing the class prediction [23]; thus, decision trees provide a unique and transparent way of inducing knowledge from data, which is different from many other classifiers working as black-box models. Specifically, three decision tree algorithms [24] were used within the framework: Classification and Regression Trees (CARTs), C5.0 trees, and Conditional Inference (CI) trees. Importantly, C5.0 tree is used as a cost-sensitive classifier to tackle the imbalance of classes during classification for good generalization on new data samples.

### 2.2. Performance Metrics

There are many different ways to evaluate the performance of a machine-learning classifier. For imbalanced datasets, which are very common in clinical studies, the *F*_1_*-score* is a preferred metric, as it makes more sense than others [25]. As a harmonic mean of precision (positive predictive value) and recall (sensitivity), *F*_1_*-score* uses its balanced form to assess the classifiers’ performance.

$$F_{1}\text{-}score=\frac{2\cdot recall\cdot precision}{\left(recall+precision\right)}$$

Ranking process, models implementations, and performance evaluation were all performed in RStudio version 3.0.2. 64 bits, a multiplatform, open source integrated development environment for statistical computing in R language. Packages rpart for CART trees, *C50* for C5.0 trees, and party for conditional inference trees were used within RStudio. The *caret* package was used to select the ranges for the tuning parameters for the grid search via cross-validation.

### 2.3. Datasets

We used a cohort of patients with the conditions AS and RA as exemplars of the application of the data-driven framework. RA affects a large number of people, which allows a large cohort to be identified. Previously, researchers and clinicians turned to the Quality Outcomes Framework (QOF) to identify the condition of RA. The QOF is a-pay-for-performance scheme established in UK in which a standardised set of codes is used to find the prevalence of a condition in a practice [26]. In this study, the developed algorithm was compared with the QOF-based results. AS is considerably less prevalent than RA, and the development of AS is less understood, and diagnosis can take several years to be confirmed. These differences allow the method to be tested against very different cases to better assess its effectiveness.

Data were extracted from the SAIL databank [27], which is a national e-health infrastructure for linking a wide range of person-based, health-related data schemas. This study used GP records across the Abertawe Bro Morgannwg University Health Board (ABMU) and Cardiff areas and linked to the local rheumatology secondary care clinical database-CELLMA (RioMed Ltd. UK). The CELLMA data were used as the gold standard for training models to identify patients with RA or AS. Individuals were identified as being valid if they had continuous coverage within the period between 2002 and 2012. This excluded people born after 2002 and people who had moved out of the area or died within the period of interest.

The GP data uses the 5-digit Read codes to administer patient health records that relate to diagnosis, medication, and process. The CELLMA system uses SNOMED-CT codes to record diagnosis and medications as well as clinical data entered by rheumatologists at the point of capture.

In this study, multiple sources of EHRs, including 544,537 patient records from ABMU CELLMA, 150,417 records from Cardiff CELLMA, 917,496,869 records from primary care, and 40,656,805 records from secondary care within the period between 2002 and 2012, were collected to generate the dataset. Thus, the linked dataset is comprised of a total of 9657 patients (8723 in ABMU and 934 in the Cardiff area), from which 1484 were positive in RA (ABMU: 1189—13.63% prevalence; Cardiff: 295—31.58% prevalence), and 204 were positive in AS (ABMU: 163—1.87% prevalence; Cardiff: 4.39% prevalence) (See Table 1).

### 2.4. The Data-Driven Framework for Patient Cohort Identification

**Figure 1:**
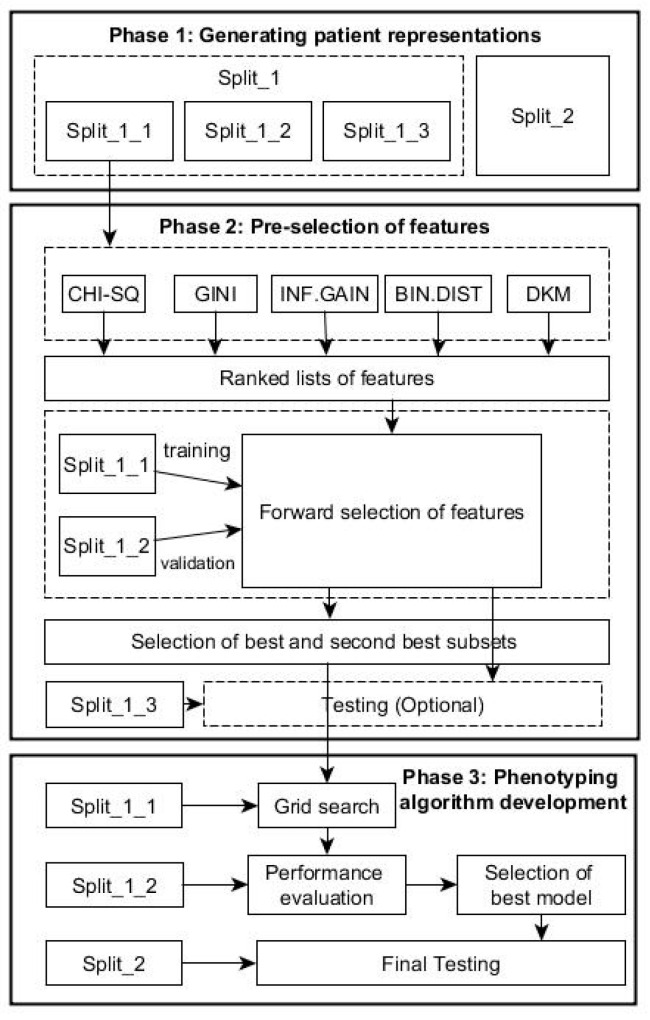
Flow chart with the phases of the data-driven framework for patient cohort identification.

To identify cohort of patients with a particular condition, we integrated the feature selection and machine-learning techniques in one framework. Specifically, the proposed data-driven framework consists of the following phases as shown in Figure 1.

#### 2.4.1. Phase 1: Generating Patient Representations

The RCT system with 5 bytes provides around 83,000 clinical descriptive terms in hierarchical structure, comprising five levels of detail with more detail to a concept in each successive level; even the SNOMED system offers much more descriptive terms than the RCT. In this study, we treated each patient as a document described by a series of coded terms and other variables (words) as in text classification, but the exact ordering of the terms was ignored, and only the number of occurrences of each term mattered (i.e., a Bag of Words). In this way, we created numeric representations of patients from EHRs by assigning each term with its frequency: how frequently the term occurs in the e-health records of a patient. In this way, each patient is treated as a vector with one component corresponding to each term.

For the proposed framework, we split the data in two parts: Split_1 and Split_2 (Table 1). The first part, Split_1, was used within both phases 2 and 3 below, while Split_2, from a population in a different geographical area, was used for evaluating the generalization performance of the final algorithm resulted from *Phase 3* below. Split 1 was subsequently divided in 3 parts with training subset Split_1 (60%) for feature ranking and constructing classifier, validation subset Split_2 (20%) for selecting optimal predictors, and testing subset Split_3 (20%) for testing the performance of the predictors in Phase 2.

#### 2.4.2. Phase 2: Pre-Selection of Features

Given *K* features, each of the *K* features can be either be selected or excluded, and as a result, there are *2^K^* possible feature subsets. This poses a model-selection challenge over *2^K^* possible models because linked medical dataset often has a large number of features, i.e., *K* is very large, while it is usually too computationally expensive to explicitly enumerate over and compare all *2^K^* models. Heuristic scheme is typically used to identify the subset of good features as described below. This phase comprises following steps:

##### Ranked Features Generation

Each feature (e.g., Read codes in our dataset) will occur in a number of times for each patient [28]. Using Split_1_1, the five FS methods described in Section 2 are used to generate five ranked lists of features in terms of their abilities to distinguish the health outcomes of patients. Assume that Ω is the feature-ranking results obtained in terms of a chosen ranking index Ω={Ω(1),Ω(2),⋯,Ω(K)}, where *K* denotes the total number of features.

##### Forward Selection of Features

After ranking features from the most significant one to the least significant one using a significance index (i.e., Ω(i) decreases as *i =* 1, …, *K*), one can use a procedure, termed as forward selection, to determine the smallest subset of features that explain the available data well. Let Σ be the features selected recursively.

**Figure 2:**
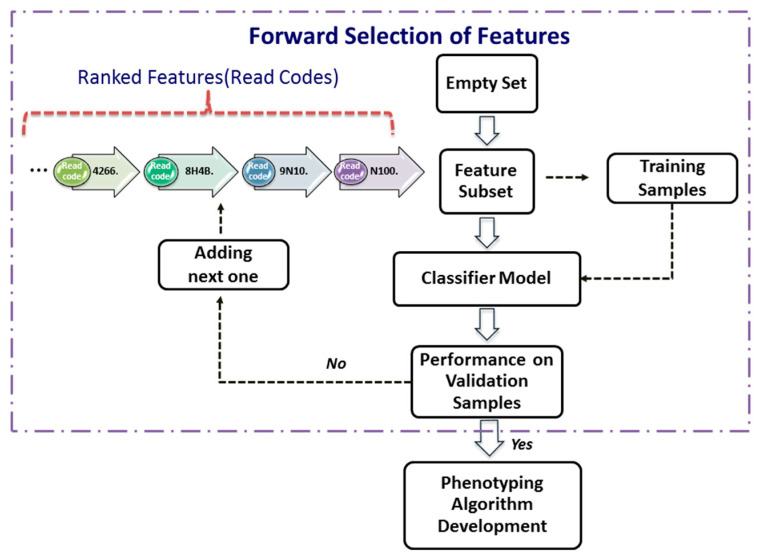
Forward selection of features.

The forward-selection procedure (Figure 2) is a heuristics for feature selection that starts with an empty set of features (i.e., Σ=∅). One at a time, the most important feature from the Ω is added to Σ, the validation error of the predictive model constructed by these features from the Split_1_1 data. This feature-selection process continues until the validation performance of the constructed model applied to the validation dataset, Split_1_2, is satisfied. As depicted in the Figure 2, the forward selection procedure consists of the following steps:

Step 1. Set $\Sigma_{0}=\emptyset ,\;i=1$, and assign a validation error tolerance threshold.

Step 2. Select the most important feature from Ω as follows:

$$\Sigma_{i}=\Sigma_{i-1}\;\cup \Omega \left(i\right)$$

where Ω(i) is the *i*th most important feature.

Step 3. Construct a classifier model with the feature set $\Sigma_{i}$.

Step 4. Apply the classifier model to the validation dataset.

Step 5. If the validation performance is satisfied, then use this compact feature set $\Sigma_{i}$ to develop phenotyping algorithm next; otherwise, increase *i* by 1, and go to Step 2.

In this study, the model performance is assessed by its accuracy and F1-score. In order not to lose potential features, the best and the second best (highest and second highest values of accuracy and F-score) subset of features are taken to the next phase.

#### 2.4.3. Phase 3: Phenotyping Algorithm Development

This phase tries to find the optimal phenotyping algorithm based on selected feature sets. For each selected combination of features resulting from Phase 2, a grid-search is performed over different decision tree algorithm using the Split_1_1 data as the training dataset. Within this grid-search, tuning parameters for each type of decision tree described above varied until a combination in their values that results in an optimal performance was found. The performance is the overall agreement rate averaged over 10-fold across validation iterations. A more fine granularity within the ranges of parameters during the grid-search could be achieved by choosing a step increase for each parameter. For example, in this study, for the C5.0 trees, the parameter of the smallest number of samples to be put in at least two of the splits varied between 5 and 30 with a step increase of 5; the cost of misclassifying positive class into negative class varied between 1 and 20 with a step increase of 1.25. For the CART trees, the parameter of the minimum number of observations in a node in order for a split to be attempted varied between 10 and 40 with a step increase of 5, while the complexity parameter to save computing time by pruning off splits that are obviously not worthwhile varied between 0.001 and 0.1 with a step increase of 0.001. For the CI trees, the value of the test statistic or 1—the *p*-value that must be exceeded in order to implement a split—varied between 0.1 and 0.99 with a step increase of 0.1. The minimum sum of weights in a node in order to be considered for splitting varied between 35 and 45 with a step increase of 5.

After all decision trees are generated for each of the selected combinations of features, the validation dataset, Split_1_2, is used to select the tree with best performance for each of the combinations, and subsequently, the best of all trees is selected among this list. To test the performance, Split_2 is used at this stage.

### 2.5. Complexity Analysis of Selecting Set of Good Features

Traditional forward-selection procedure uses exhaustive search to identify the set of good features in which at the start, the selected set is empty, and all *K* features are candidates. This means the first iteration tests *K* candidates, the second *K*−1, …, etc., which leads to K(K+1)/2 runs of classification models to determine good features. In this paper, we first evaluated the importance of each feature and ranked them using feature ranking index, then the forward selection first evaluated the most important feature, the second most important feature, …, etc., until the set of selected features could achieve the best classification performance on validation samples (let *k* be the number of selected features in the feature set). In other words, we did not exhaustively search all feature candidates; instead, we only ran *k* times of classification models. However, the computational cost of feature ranking needs to be taken into account, for example, the computational complexity of calculating original χ^2^ for a feature is *O(I × J)* [29] using a contingency table with *I* rows and *J* columns. Gini-index-based feature selection is bounded by O(K *N_trn_ + K logK) [30], where *N_trn_* is the number of training samples. The running cost of calculating information gain is *O(K·c)* [31], where c is a number of classes (*c = 2* in this paper). The time complexity of using dynamic programming approach to implement the binomial coefficient *C(K, k)* is O(*K·k*) [32].

Additionally, the full complexity of feature selection also depends on the computational cost of classifier. For example, this study used the decision tree C5.0 model with complexity of *O(h · l · (c · N_trn_ + N_trn_ · log N_trn_))* [31], where *l* is the number of features in the tree, and *h* is a tree height. Considering the C5.0 model was run *k* times, from a model with 1 feature, a model with 2 features … to a model with *k* features, we can calculate the full complexity of feature selection as

(1) $$O\left(\max\left(R_{t},h\cdot k^{2}\left(c\cdot N_{trn}log\cdot N_{trn}\right)\right)\right)$$

where *R_t_* is the cost of feature ranking as decribed above. This computational cost is much better than that of traditional forward selection scheme $O\left(h\cdot K^{2}\left(c\cdot N_{trn}log\cdot N_{trn}\right)\right)$, in which the classifier is C5.0 model and K≫k. In other words, the proposal feature selection framework is much more computationally efficient.

## 3. Results

### 3.1. Results by the Data-Driven Framework

In Phase 1, the Bag of Words scheme in text mining led to a complete clinical dataset with a total of 27,293 different single codes, which means a patient-term matrix of 9657 × 27,293 dimensions was created to represent patient profiles. As described above, this dataset was further split into the subsets of Split_1_1, Split_1_2, and Split_1_3 for feature selection and Split_2 for evaluating the generalisation performance of optimal phenotyping algorithm in grid-search.

In Phase 2, the 27,293 different clinical codes were ranked separately by each of the five FS methods. Table 2 and Table 3 show, for the RA and AS datasets, respectively, the best and second best combination of features selected for every FS method after applying the forward selection procedure (Figure 2). The tables show the two best results for accuracy and F_1_-score obtained for each method that were used to the parsimonious sets of features needed for the next phase. The tables also show the best and second best combinations for each method on testing samples. The χ2 and Gini were presented together, as they selected the same predictors.

For RA, the most compact set of features was obtained by the χ^2^ and Gini methods. Comparatively, to obtain similar performance, it was necessary to select 234 features by IG method, or 238 features by Binomial method, or 263 features by the DKM method because similar predictors were selected, and therefore, they gave best and second-best combinations with similar total number of features. In the case of AS, promisingly, the most compact sets with two features by χ2 and Gini, or IG methods, or three features by Binomial method were identified. The DKM index ranked features differently than other methods, and larger number of features were identified.

In Phase 3, the two best-selected lists of features given by each method were taken to Phase 3. The best performance for the RA dataset was achieved by using the C5.0 decision tree with the second list selected by the Binomial distribution method. Similar performance with fewer features was achieved with the C5.0 tree and the first list of features selected by the DKM index. For the AS dataset, the best decision tree with fewer features resulted from the C5.0 and CART algorithms with the first feature list selected by the Binomial distribution. C5.0 and CART trees obtained marginally better performance with the second feature list selected by the IG method.

In both datasets, the CI trees gave a worse performance than C5.0 and CART trees. Performance of all decision trees, selected best algorithms, and corresponding tuning parameters are shown in the Appendix A. Appendix B illustrates list of Read codes appearing in the decision trees and corresponding description, in which Table A1 shows the Read codes appearing in the decision trees for AS.

### 3.2. Comparison with Human Knowledge-Driven Algorithms

#### 3.2.1. The QOF-Based Ruleset for Rheumatoid Arthritis

We compared the proposed data-driven methodology with the QOF-based ruleset in identifying RA patients [33]. The QOF uses a set of Read codes to determine whether a patient has an RA diagnosis. These codes are included in the Table A2 (Appendix B). Table 4 shows the performance achieved by using the QOF rules on the Cardiff dataset (Split_2) and the two best decision trees obtained with the first feature list selected by the DKM index (DKM-1) and the second feature list selected by the Binomial distribution (BIN-2). It can be seen that, overall, the DKM-1 algorithm with the accuracy of 86.19% performed slightly better than the QOF rules with 85.85%. However, the QOF rules obtained better positive predictive value (PPV) (85.28%) than DKM-1 algorithm, while the DKM-1 achieved higher sensitivity (72.20%) than the QOF rules (66.78%). The BIN-2 had better performance in terms of specificity (95.77%) and PPV (87.32%).

#### 3.2.2. The Clinical Standard Procedure for Ankylosing Spondylitis

There are no QOF rulesets defined for AS. The standard method followed by clinicians to identify the prevalence of AS in a population is simply using the Read code “*N100*.”. Applied to our dataset, this standard method achieved 95.65% PPV and 97.86% accuracy (Table 5). However, this resulted in a low sensitivity (53.66%), which means that almost half of the positive cases would remain unidentified. Promisingly, our data-driven algorithm (IG-2) also identified “*N100*.” as the most important feature in identifying AS, together with two additional codes, “F440.” and “k65z.” corresponding to diagnosis and treatment of uveitis, a condition that has been strongly related to AS [34].

## 4. Discussion

By selecting the most relevant features from a huge number of primary care codes, this framework can bring multiple benefits to health informatics research. Firstly, dimensionality reduction makes many machine-learning classifiers feasible for large data problems due to avoiding the curse of dimensionality. Secondly, using only the most relevant codes (terms) can significantly speed up the training and testing process of the classifiers, which makes the development of a phenotyping algorithm effective. Thirdly, using those relevant codes can remove the noises and reduce biases that distort the true relationship between the risk factors and the outcome. Fourthly, using only the most relevant features can avoid the over-fitting dilemma, a common challenge in the development of data-driven approaches, which would help improve the generalisation performance of the classifiers applied to un-seen patients’ data. Importantly, with only the most relevant features, the developed classifiers themselves and their prediction results would achieve better transparency and interpretation in clinical decision supports [15,33,34,35,36].

On the other hand, chronic diseases, like RA and AS, often take years to develop and be properly diagnosed. For instance, AS can take more than 10 years to be diagnosed, and when a formal diagnosis is made, the disease is in a very advanced state [37]. The proposed method is used in retrospective to extract knowledge from the medical history of already diagnosed AS patients and therefore discover early symptoms of AS. In this way, this framework provides an efficient option for identifying cohort of patients with a particular condition, especially where knowledge-driven approaches, such as QOF-based rulesets, do not yet exist. In addition, by learning directly from the data, the data-driven algorithm generated by the proposed framework might potentially reduce bias and/or errors derived from applying other algorithms implemented using a specific population with different characteristics.

For RA, the algorithms development discovered a set of codes related to drugs that are commonly given to RA patients [38]. The particular Read codes identified by the data-driven method may be specific characteristics of the studied population across ABMU and Cardiff regions. Reasonably, such a unique set of codes may vary for other populations (datasets). Promisingly, the proposed framework was demonstrated to identify these unique sets of codes related to certain populations from given datasets.

Our results indicate that the χ^2^ statistic and the Gini index generated the same ranking of features in both datasets. This is mainly because the χ^2^ statistic coefficient for a 2 by 2 contingency table can be also interpreted as the Gini coefficient [39]. Each feature-selection method works differently depending on the nature of the data. To identify patients with a particular profile, a single feature-section method may generate incomplete results. Our recommendation is to use multiple feature-selection methods to generate a bigger picture of prediction performances and select an optimal algorithm with consideration of clinical practices.

We stress that the proposed framework aiming at identifying influential features actually fulfils the task of dimensionality reduction in nature. In a high-dimensional data space, dimensionality reduction can be performed by two different schemes. One scheme is to directly select influential features from original features, in which the selected features work together to demonstrate good prediction performance, such as our proposed framework. The other scheme is to transform all original features into a low-dimensional space, in which each dimension of this space works as a latent variable while holding certain statistical property of original features, such as the principal component analysis. Each scheme has its own advantages and disadvantages, but in disease phenotyping and patient identification, the first scheme is preferred. This is mainly because the first scheme can maintain the physical meanings of original variables in a low-dimensional space so that the finally selected features in this space can gain clear interpretations in prediction. The second scheme uses latent variables in a low-dimensional space that are often difficult for which to gain clear physical meanings.

As a baseline method for identifying optimal hyper-parameters, grid-search can provide coarse but uniform exploration of the parameter space. The performances of decision tree models varied greatly for different values of the parameters. The C5.0 trees are very sensitive to the two parameters of minimum cases for split and asymmetric cost. For the RA and AS, we found that the optimal parameters are particular to each dataset. That is to say, one cannot expect a single optimal parameter set can be generalised to different datasets. In the future, heuristic optimisation procedures will be used to reduce the computing loads in identifying optimal hyper-parameters.

Medical data sets are often predominately composed of “normal” cases (a large number of population without a condition of interest) together with a small percentage of “abnormal” cases (a small number of cases with this condition of interest, such as RA or AS). Such an imbalance presents methodological challenges in developing and verifying machine-learning algorithms. This study used the C5.0 tree model to tackle imbalanced classification problems by benefiting from the asymmetric costs implemented in majority class and minority class.

## 5. Conclusions

We have presented a transparent machine-learning framework capable of identifying cohorts of people with certain disease from EHR in primary care. We have validated this framework for two chronic diseases, RA and AS. We have demonstrated that the machine-learning framework performed as well as the existing clinical-knowledge-driven approach. This study provides an efficient way of identifying cohort of patients with a particular condition in primary care. The proposed framework has a promising potential of being widely used in health-related studies, such as clinical trial recruitments, survival analysis, or outcome predictions, etc.

## Figures and Tables

**Table 1:** Datasets.

		Rheumatoid Arthritis		Ankylosing Spondylitis	
		0	1	0	1
**COMPLETE DATASET**		8173	1484	9453	204
**ABMU data**	**Complete ABMU**	7534	1189	8560	163
	**Training (Split_1_1)**	4521	713	5133	101
	**Validation (Split_1_2)**	1515	230	1709	36
	**Testing (Split_1_3)**	1498	246	1718	26
**CARDIFF data**	**Testing (Split_2)**	639	295	893	41

**Table 2:** Pre-selection of Features for Rheumatoid Arthritis.

Feature Selection Method	Cut-Off Point	Compact Set of Features Selected	Acc. (Val.)	Acc. (Test)	F_1_-Score (Val.)	F_1_-Score (Test)
**χ2/Gini**	**First**	125	92.67	93.12	0.73	0.75
	**Second**	191	92.43	93.06	0.72	0.75
**IG**	**First**	234	92.5	93.12	0.72	0.75
	**Second**	300	92.5	93.12	0.72	0.75
**Binomial**	**First**	238	92.5	93.12	0.72	0.75
	**Second**	310	92.5	93.12	0.72	0.75
**DKM**	**First**	263	92.5	93.12	0.72	0.75
	**Second**	347	92.5	93.12	0.72	0.75

Acc., accuracy; Val., validation.

**Table 3:** Pre-selection of Features for Ankylosing Spondylitis.

		Ankylosing Spondylitis Dataset				
Feature Selection Method	Cut-Off Point	Compact Sets of Features Selected	Acc. (Val.)	Acc. (Test)	F_1_-Score (Val.)	F_1_-Score (Test)
**χ2/Gini**	**First**	2	99.08	99.48	0.75	0.82
	**Second**	306	97.94	98.45	0.57	0.61
**IG**	**First**	2	99.08	99.48	0.75	0.82
	**Second**	74	97.19	97.36	0.51	0.48
**Binomial**	**First**	3	99.08	99.48	0.75	0.82
	**Second**	81	97.19	97.42	0.51	0.48
**DKM**	**First**	138	97.19	97.42	0.51	0.48
	**Second**	293	97.02	97.65	0.48	0.51

Acc., accuracy; Val., validation.

**Table 4:** The Two Best Data-Driven Models Compared with QOF Rules for RA.

	RA Algorithms		
Performance	QOF Rules for RA	DKM-1 Algorithm	BIN-2 Algorithm
**Accuracy**	85.85	86.19	85.44
**Sensitivity**	66.78	72.2	63.05
**Specificity**	94.67	92.64	95.77
**Positive Predictive Value**	85.28	81.92	87.32
**Negative Predictive Value**	86.04	87.83	84.88

**Table 5:** The Best Data-Driven Model Compared to Clinical Method for AS.

	AS Algorithms	
Performance	Clinical Procedure (N100)	IG-2 Algorithm
**Accuracy**	97.86	97.75
**Sensitivity**	53.66	56.1
**Specificity**	99.89	99.66
**Positive Predictive Value**	95.65	88.46

**Figure A1:**
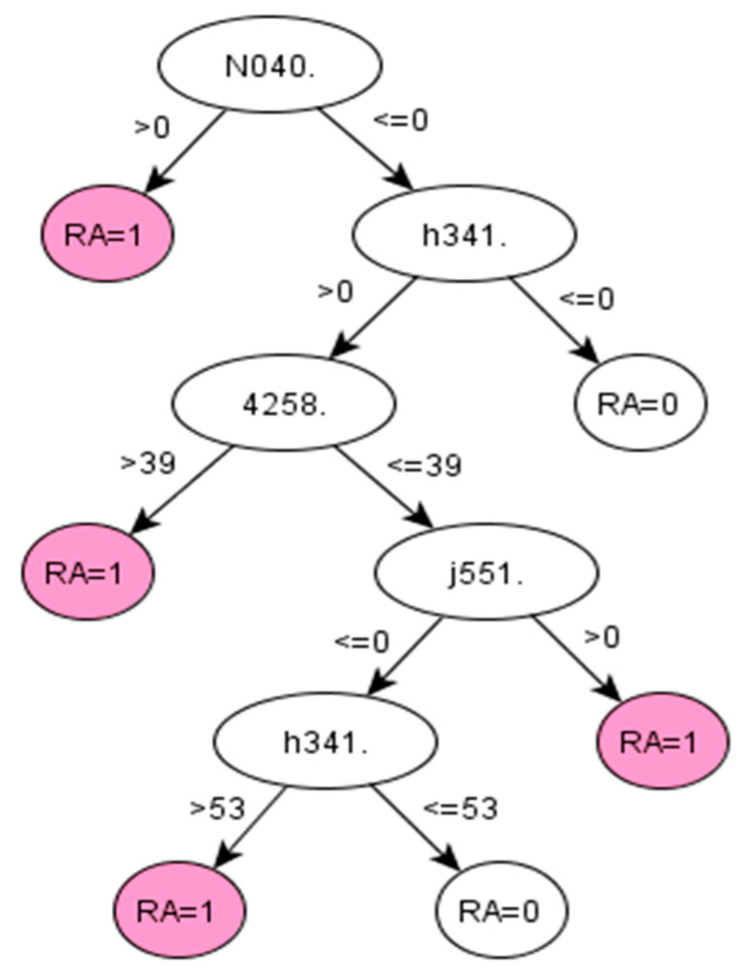
C5.0 decision tree 1 for Rheumatoid Arthritis: Initial number of features = 263; Minimum cases for split = 20; Winnowing (pre-selection of features) = True; Asymmetric cost (preference to positive class) = 1.5.

**Figure A2:**
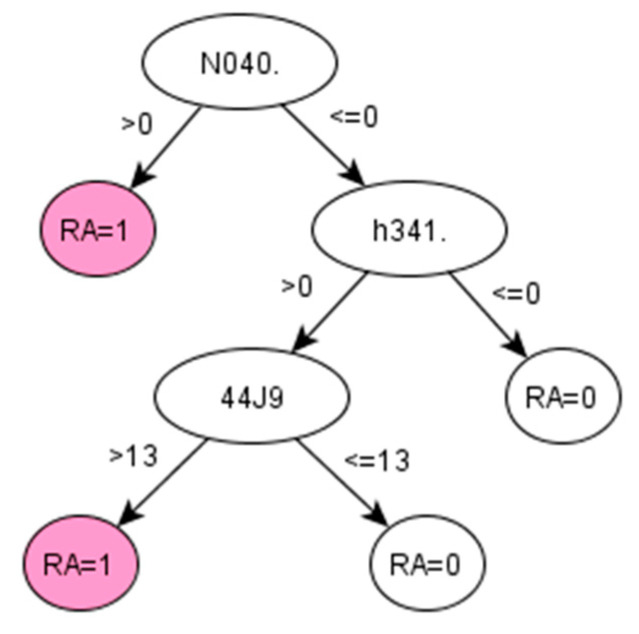
C5.0 decision tree 2 for Rheumatoid Arthritis: Initial number of features = 310; Minimum cases for split = 20; Winnowing (pre-selection of features) = True; Asymmetric cost (preference to positive class) = 2.

**Figure A3:**
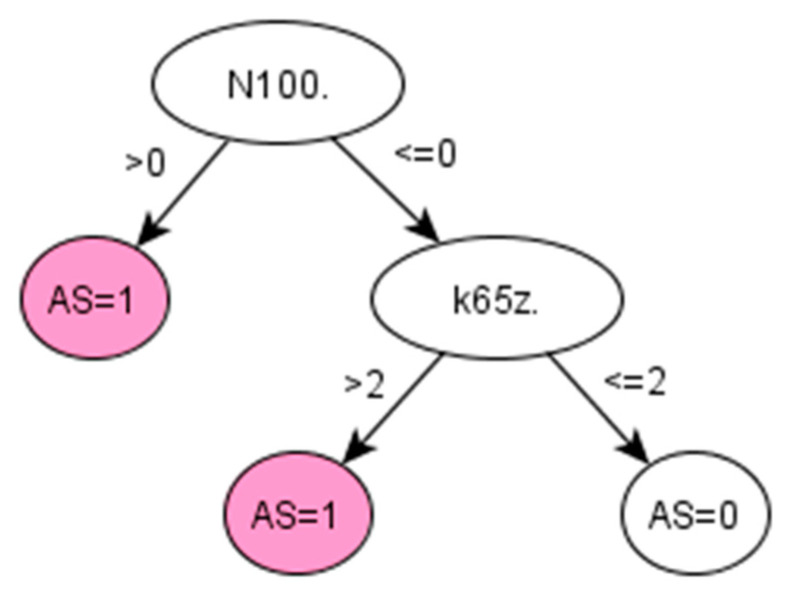
Decision tree 1 for Ankylosing Spondylitis: Initial number of features = 3; Minimum cases for split = 10; Winnowing (pre-selection of features) = False; Asymmetric cost (preference to positive class) = 15.

**Figure A4:**
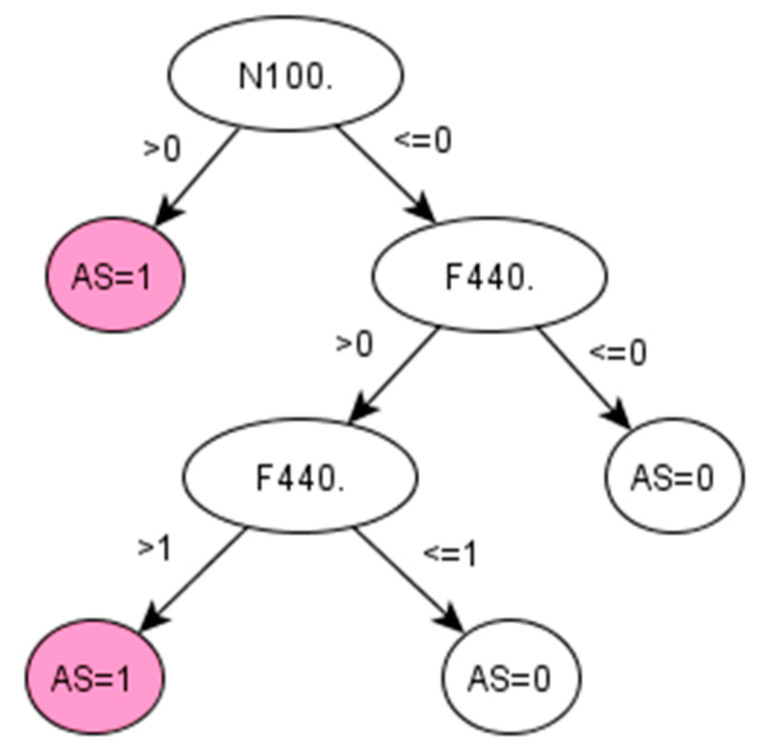
Decision tree 2 for Ankylosing Spondylitis: Initial number of features = 74; Minimum cases for split = 15; Winnowing (pre-selection of features) = False; Asymmetric cost (preference to positive class) = 6.2.

## Data Availability

Data are available from the SAIL (Secure Anonymised Information Linkage) Databank for researchers who meet the criteria for access to confidential data.

## Appendix A

Best Decision Trees Resulting from the Data-Driven Framework Applied to the RA and AS Datasets.

## Appendix B

List of Read codes appearing in the decision trees and corresponding description

**Table A1:** Read codes appearing in the decision trees for AS.

Read Codes	Type	Description
N040.	Musculoskeletal and connective tissue disease	Rheumatoid arthritis
h341.	Chemotherapy/Immunosuppressant drugs	METHOTREXATE 2.5 mg tablets
4258.	Laboratory procedures	Haematocrit
j551.	Musculoskeletal drugs	SALAZOPYRIN EN 500 mg e/c tablets
44J9.	Laboratory procedures	Serum urea level
N100.	Musculoskeletal and connective tissue disease	Ankylosing spondylitis
k65z.	Eye drugs	FLUOROMETHOLONE eye drops
F440.	Nervous system and sense organ diseases	Acute and subacute iridocyclitis

**Table A2:** QOF given Read Codes to identify prevalence of RA.

READ CD	Description
N040.	Rheumatoid Arthritis
N0400	Rheumatoid arthritis of cervical spine
N040T	Flare of rheumatoid arthritis
N0402	Rheumatoid arthritis of shoulder
N040S	Rheumatoid arthritis - multiple joint
N0409	Rheumatoid arthritis of PIP joint of finger
N0408	Rheumatoid arthritis of MCP joint
N0401	Other rheumatoid arthritis of spine
N0400	Rheumatoid arthritis of cervical spine
N0407	Rheumatoid arthritis of wrist
N040B	Rheumatoid arthritis of hip
N040D	Rheumatoid arthritis of knee
N040K	Rheumatoid arthritis of 1st MTP joint
N040F	Rheumatoid arthritis of ankle
N0405	Rheumatoid arthritis of elbow
N040A	Rheumatoid arthritis of DIP joint of finger
N0406	Rheumatoid arthritis of distal radio-ulnar joint
N040H	Rheumatoid arthritis of talonavicular joint
N040J	Rheumatoid arthritis of other tarsal joint
N040G	Rheumatoid arthritis of subtalar joint
N040L	Rheumatoid arthritis of lesser MTP joint
N040C	Rheumatoid arthritis of sacro-iliac joint
N0404	Rheumatoid arthritis of acromioclavicular joint
N040N	Rheumatoid vasculitis
N040R	Rheumatoid nodule
N040P	Seronegative rheumatoid arthritis
N041.	Felty’s syndrome
N042.	Other rheumatoid arthropathy with visceral or systemic involvement (Excluding N0420 Rheumatic carditis (v27))
N0422	Rheumatoid nodule
N042z	Rheumatoid arthropathy + visceral/systemic involvement NOS
N0421	Rheumatoid lung disease
N047.	Seropositive errosive rheumatoid arthritis
N04X.	[X]Seropositive rheumatoid arthritis, unspecified
N04y0	Rheumatoid lung
N04y2	Adult onset Still’s disease
Nyu11	[X]Other seropositive rheumatoid arthritis
Nyu12	[X]Other specified rheumatoid arthritis
Nyu1G	[X]Seropositive rheumatoid arthritis, unspecified
Nyu10	[X]Rheumatoid arthritis+involvement/other organs or systems
G5yA.	Rheumatoid carditis
G5y8.	Rheumatoid myocarditis
